# Fuchs heterochromic iridocyclitis-associated glaucoma: a retrospective comparison of primary Ahmed glaucoma valve implantation and trabeculectomy with mitomycin C

**DOI:** 10.12688/f1000research.15244.2

**Published:** 2018-10-15

**Authors:** Hamed Esfandiari, Nils A. Loewen, Kiana Hassanpour, Ali Fatourechi, Shahin Yazdani, Chao Wang, Mehdi Yaseri, Mohammad Pakravan

**Affiliations:** 1 Ophthalmic Research Center, Shahid Beheshti University of Medical Sciences, Tehran, Tehran, 19839-63113, Iran; 2 Department of Ophthalmology, School of Medicine, University of Pittsburgh, Pittsburgh, Pennsylvania, 15213, USA; 3 Ocular Tissue Engineering Research Center, Shahid Beheshti University of Medical Sciences, Tehran, Tehran, 19839-63113, Iran; 4 Department of Ophthalmology, Xiangya Hospital, Central South University, Changsha, Hunan, 410008, China; 5 Department of Epidemiology and Biostatistics, School of Public Health, Tehran University of Medical Sciences, Tehran, Tehran, 19839-63113, Iran

**Keywords:** Fuchs heterochromic iridocyclitis; glaucoma drainage implant; trabeculectomy; uveitic glaucoma.

## Abstract

**Background:** The aim of this study was to compare the safety and efficacy of primary trabeculectomy with mitomycin C and Ahmed glaucoma valve (AGV) implantation in patients with Fuchs heterochromic iridocyclitis (FHIC)-related glaucoma, a rare complication of an uncommon form of uveitis.

**Method**s **:** In this retrospective comparative case series, 26 FHIC-associated glaucoma patients received trabeculectomy (n=12) or an AGV (n=14). Primary outcome measures were surgical success, defined as intraocular pressure (IOP) ≤21 mmHg, decreasing ≥20% from baseline, and no secondary glaucoma surgery. Secondary outcome measures were the number of glaucoma medications, complications, best corrected visual acuity (BCVA), and IOP.

**Results: **The follow-up was 34.0±17.7 months in patients that received trabeculectomy and 33.4±18.6 months in AGV (P= 0.837). The cumulative probability of success rate was 41.7% for trabeculectomy and 85.7% for AGV, with no significant difference in complications (P>0.05). The IOP in patients that received trabeculectomy dropped from 23.4±3.3 mmHg to 21.6±5.2 mmHg at the final visit (P= 0.041). In patients that received AGV, the IOP decreased from 24±7.8 to 17.1±2.6 mmHg (P= 0.003). The number of glaucoma medications at baseline were 3.3±0.5 in those that received trabeculectomy and 3±0.6 in those that received AGV (P=0.233), and decreased to 2.4±1.0 (P=0.008) and 1.7±0.6 (P=0.002), respectively. BCVA was equal in both groups and did not change (P>0.05).

**Conclusion:** Primary AGV had a higher success rate than trabeculectomy, with patients also needing fewer medications for the management of FHIC-associated glaucoma.

## Introduction

Fuchs heterochromic iridocyclitis (FHIC) is a rare form of uveitis. While the incidence of all forms of uveitis is approximately 0.035% of the population ^1^, the incidence of FHIC is only about 0.00105% (3% of all uveitis cases) ^2, 3^ and occurs in both eyes in 10% of patients ^4^. It is characterized by low-grade intraocular inflammation, small stellate keratic precipitates, and iris stromal atrophy ^5^. Recent evidence points towards an association between rubella and FHIC ^6, 7^, but an association between FHIC and toxoplasmosis and toxocariasis has also been reported ^8, 9^. Affected patients are often asymptomatic for years and mostly present with symptoms of a cataract or floaters during the third or fourth decade of life. Because the presentation is often variable, FHIC is among the most underdiagnosed conditions in ophthalmology ^10^. Since there is an average 3.7-year delay in diagnosing FHIC, it should be considered as a differential diagnosis for any young patient with unilateral low-grade uveitis and good visual acuity ^5^. Although FHIC is frequently complicated by cataract formation in two-thirds of patients, the outcome of phacoemulsification and intraocular lens implantation is excellent and comparable to that in normal eyes ^11^. Older age and a cataract can put patients with FHIC at risk of glaucoma ^12^ which occurs in 15 to 59% ^13, 14^.

Since anterior and posterior synechiae are uncommon in this condition, angle-closure mechanisms do not play an important role in the development of glaucoma. Abnormal angle vessels, physical obstruction of trabecular meshwork by inflammatory cells, disruption of uveal and juxtacanalicular structures, trabecular meshwork fibrosis and steroid-induced ocular hypertension are all contributing causes ^13, 15^.

FHIC often responds poorly to medical management, requiring a surgical intervention to control intraocular pressure (IOP) ^11, 13, 14, 16^. There is a paucity of literature regarding the best initial surgical approach in the management of FHIC-associated glaucoma. The purpose of this study was to compare the outcomes of the two most common surgical interventions, glaucoma drainage device implantation, and trabeculectomy, for glaucoma caused by FHIC.

## Methods

### Subject selection, demographics and outcomes

This study was approved by the ethics committee and the Institutional Review Board (IRB) at the Ophthalmic Research Center of Shahid Beheshti University of Medical Sciences (Tehran, Iran, protocol number: IR.SBMU.ORC.REC.1391.2) and followed the tenets of the Declaration of Helsinki. The IRB waived patient consent for the use of their medical records in this retrospective chart review. The chart review occured at the Labbafinejad Medical Center, Tehran, Iran, and included charts from May 2001 to September 2017, yielding 26 patients with FHIC-associated glaucoma that either had mitomycin C (MMC)-augmented trabeculectomy or a primary Ahmed glaucoma valve (AGV) implantation. Inclusion criteria were age equal to or above 18 years of age and a diagnosis of FHIC-associated glaucoma. FHIC-associated glaucoma was defined as cases of previously known FHIC or diagnosed as FHIC at the time of presentation accompanied by uncontrolled IOP and progressive glaucomatous optic neuropathy. Exclusion criteria consisted of prior glaucoma surgery, ocular or systemic comorbidities that could affect the procedure and study outcomes including immunodeficiency, connective tissue disease and uncontrolled diabetes. Patients were not formally matched across demographics.

Demographic and baseline data, including age, gender, baseline best corrected visual acuity (BCVA), IOP, number of medications, anterior chamber cells ^17^, type of surgery, and surgical details were recorded. In all cases, surgery was only performed when the eyes were not more than 0.5 inflamed ^18^. Primary outcome measures were surgical success defined as IOP ≤21 mmHg and decreased ≥20% from the baseline, no secondary glaucoma surgery, and no loss of light perception.

Secondary outcome measures were the rate of complications, cataract development, number of medications, IOP reduction and inflammation. Hypotony was defined as an IOP <6 mmHg at any postoperative visit, and hypertensive phase following AGV implantation was defined as an IOP >21 mmHg during the first 3 months after the surgery (with or without medications) after achieving IOP less than 21 after the surgery ^19^. All postoperative data for each surgery were documented until the last follow-up visit or when a secondary glaucoma surgery was performed. Three surgeons performed the surgeries and patients received each surgery based on the surgeon’s preference and comfort as well as patients’ decision.

### Surgical techniques

***Trabeculectomy***. A 7-0 silk traction suture was passed through the superior cornea. A conjunctival peritomy was performed at the supranasal quadrant followed by Tenon’s dissection. Wet-field cautery was used to stop episcleral vessels bleeding. A 4×3 mm trapezoidal half-thickness scleral flap was created, followed by lamellar dissection to the peripheral cornea. Sponges soaked in 0.04% MMC were applied for 3 minutes. After creating a sideport, a keratome was used to enter the anterior chamber underneath the flap, and a block of clear cornea was removed using a Kelly punch. The scleral flap was closed relatively tightly with two releasable sutures so that spontaneous drainage was minimal. The conjunctiva was closed with 10-0 nylon sutures. At the conclusion of surgery betamethasone and cefazolin were injected into the subtenon space away from the site of operation. The postoperative regimen consisted of chloramphenicol 0.5% eye drops (Sina Darou Lab. Co., Tehran, Iran) four times a day for 1 week and betamethasone 0.1% eye drops (Sina Darou Lab. Co., Tehran, Iran) six times a day, which was tapered to 4, 3, 2, 1 times a day every two weeks.

***Ahmed glaucoma valve implantation***. A 7-0 silk traction suture was placed through the superior clear cornea. The conjunctiva was opened 4 mm posterior to the limbus in the supratemporal quadrant, and a blunt dissection of the Tenon was performed using Westcott scissors to provide space for the plate insertion. The device (Ahmed glaucoma drainage implant, model FP7, New World Medical, Rancho Cucamonga, CA, USA) was primed with 2 ml of buffered saline solution (BSS) and gently pushed into the subtenon space. The plate was secured to the sclera 10 mm posterior to the limbus using 7-0 silk sutures. The tube was trimmed bevel-up with an estimated intracameral length of 2 mm. A 23-gauge needle was inserted into the anterior chamber bevel-up, parallel to the iris and 1 mm posterior to the limbus. The tube was passed through the tunnel into the anterior chamber and secured to the sclera with a 10-0 nylon suture. A 5×8 mm scleral patch graft was placed over the tube. Tenon’s capsule and the conjunctiva were closed using a running 10-0 nylon mattress suture. At the end of the surgery, 0.5 ml of subtenon triamcinolone (40 mg/ml) was injected next to the plate in four patients. Betamethasone (4 mg) and cefazolin (50 mg) were injected into the inferior subconjunctival space upon conclusion of the surgery. The postoperative regimen consisted of chloramphenicol 0.5% eye drops four times a day for 1 week and betamethasone 0.1% eye drops six times a day, which was tapered to 4, 3, 2, 1 times a day every two weeks.

### Statistics

To test for a difference between the two groups at baseline, we used the t-test, Mann-Whitney, chi-square and Fisher’s exact test. We used a general linear model and ordinal logistic regression to compare the groups adjusted for the baseline. Changes within groups were evaluated using paired t-test and Wilcoxon signed rank test. A P-value less than 0.05 was considered statistically significant. All statistical analyses were performed with SPSS software (IBM Corp. Released 2016. IBM SPSS Statistics for Windows, Version 24.0. Armonk, NY) Data was described as frequency (percent), mean ± standard deviation or median and range.

## Results

A total of 26 patients were included for the final analysis, of whom 14 were male (53.8%). There were 12 trabeculectomies and 14 AGV surgeries. All cases were primary surgeries with no history of glaucoma surgery. There was no significant difference regarding sex, age, IOP, BCVA, and numbers of glaucoma medications at baseline ( Table 1). The mean age at the time of surgery for trabeculectomy was 47.5±6.1 years and for AGV was 45.9±9.3 years (P= 0.608). In total, 10 patients (83.3%) in the trabeculectomy group were phakic and 14 patients (100%) in the AGV group were phakic (P=0.203). Preoperatively, the angle was open in all patients upon gonioscopy. In the trabeculectomy group, two patients had a phacoemulsification and lens implantation in the same session. The mean follow-up time was 34±17.7 months in the trabeculectomy group and 33.4±18.6 months in the AGV group (P= 0.837).

**Table 1.** Baseline clinical characteristics of patients in the trabeculectomy and Ahmed glaucoma valve (AGV) groups.

Variable		All	Trabeculectomy	AGV	P-value
Age	Mean ± SD	46.6 ± 7.9	47.5 ± 6.1	45.9 ± 9.3	0.608 ^†^
	Median (range)	48 (32–60)	48.5 (36–56)	47 (32–60)	
Gender	Male	14 (53.8%)	8 (66.7%)	6 (42.9%)	0.267 *
	Female	12 (46.2%)	4 (33.3%)	8 (57.1%)	
Lens status	Phakic	24 (92.3%)	10 (83.3%)	14 (100.0%)	0.203 **
	Pseudophakic	2 (7.7%)	2 (16.7%)	0 (0.0%)	
BCVA	Mean ± SD	0.29 ± 0.3	0.3 ± 0.34	0.27 ± 0.27	0.835 ^‡^
	Median (range)	0.15 (0.05–1.1)	0.15 (0.05–1)	0.19 (0.05–1.1)	
IOP	Mean ± SD	23.7 ± 6	23.4 ± 3.3	24 ± 7.8	0.801 ^†^
	Median (range)	22 (14–42)	23 (18–28)	21 (14–42)	
Medications	Mean ± SD	3.1 ± 0.5	3.3 ± 0.5	3 ± 0.6	0.233 ^‡^
	Median (range)	3 (2–4)	3 (3–4)	3 (2–4)	
Follow-up	Mean ± SD	33.7 ± 17.8	34 ± 17.7	33.4 ± 18.6	0.837 ^‡^
	Median (range)	29.5 (11–89)	30 (11–80)	29.5 (13–89)	

BCVA, best corrected visual acuity; IOP, intraocular pressure. †Using t-test. ‡Using Mann–Whitney U-test. *Using chi-squared test. **Using Fisher’s exact test.

Surgical success at the final follow-up was 41.7% for trabeculectomy surgery and 85.7% in AGV (P= 0.025). IOP decreased significantly from 24±7.8 mmHg at baseline to 17.14±2.6 mmHg at the final follow-up in AGV (P= 0.003). The corresponding numbers for trabeculectomy were 23.4±3.3 and 21.58±5.2 mmHg, respectively (P= 0.041; Table 2). AGV had a significantly lower average IOP at the final follow-up visit compared to trabeculectomy (P= 0.018). There were three patients in the trabeculectomy group and one in the AGV group that needed a surgical revision specifically to control high IOP. AGV was used as a secondary glaucoma surgery in all these cases. The number of glaucoma medications decreased significantly from 3±0.6 at baseline to 1.71±0.6 at the final follow-up visit in the AGV group (P= 0.002). The medications in the trabeculectomy group were 3.3±0.5 at baseline and 2.41±1.01 at the conclusion of the study, respectively (P= 0.008). Eight cases needed bleb needling with MMC injection after trabeculectomy. Needling was done during the postoperative period for impending failure from a contracting bleb. Thirty minutes after injecting 0.1 ml of 0.02% Mitomycin-C into the bleb-adjacent subtenon space a 27-gauge needle was used to reform the bleb and dissect adhesions at the slit lamp.

**Table 2.** Change of examined variables during the course of the study.

Parameter		Group		Difference	95% CI		P-value
		T	AGV		Lower	Upper	
BCVA	Baseline	0.3 ± 0.34	0.27 ± 0.27	0.03	-0.21	0.28	0.835 ‡
	Final visit	0.34 ± 0.35	0.44 ± 0.67	−0.1	-0.55	0.34	0.417 §
	Change	−0.03 ± 0.08	−0.17 ± 0.57	0.14	-0.21	0.48	
	Within P □	0.006	0.004				
IOP	Baseline	23.4 ± 3.3	24 ± 7.8	−0.6	-5.6	4.4	0.801 †
	Final visit	21.58 ± 5.2	17.14 ± 2.6	0.6	-2.4	3.6	0.018 §
	Within P $	0.041	0.003				
Medication	Baseline	3.3 ± 0.5	3 ± 0.6	0.3	-0.2	0.7	0.233 ‡
	Final visit	2.41 ± 1.01	1.71 ± 0.6	0.2	-0.5	1	0.041 ¥
	Within P □	0.008	0.002				
Baseline lens status	Phakic	10 (83.3%)	14 (100.0%)	16.70%	10.4%	−4.7%	0.203 *
	Pseudophakic	2 (16.7%)	0 (0.0%)				
Final visit lens status	Phakic	3 (25.0%)	13 (92.9%)	67.90%	14.3%	38.3%	0.001 *
	Pseudophakic	9 (75.0%)	1 (7.1%)				
Success rate at final visit		5 (41.7%)	12 (85.7%)	−22.60%	19.9%	−63.7%	0.025 *

†Using t-test. ‡Using Mann–Whitney U-test. *Using Fisher’s exact test. § Adjusted for the baseline, based on General linear model. ¥Adjusted for the baseline, based on ordinal logistic regression. □Using Wilcoxon signed rank test. $ Using paired sample t-test.

**Figure 1.**
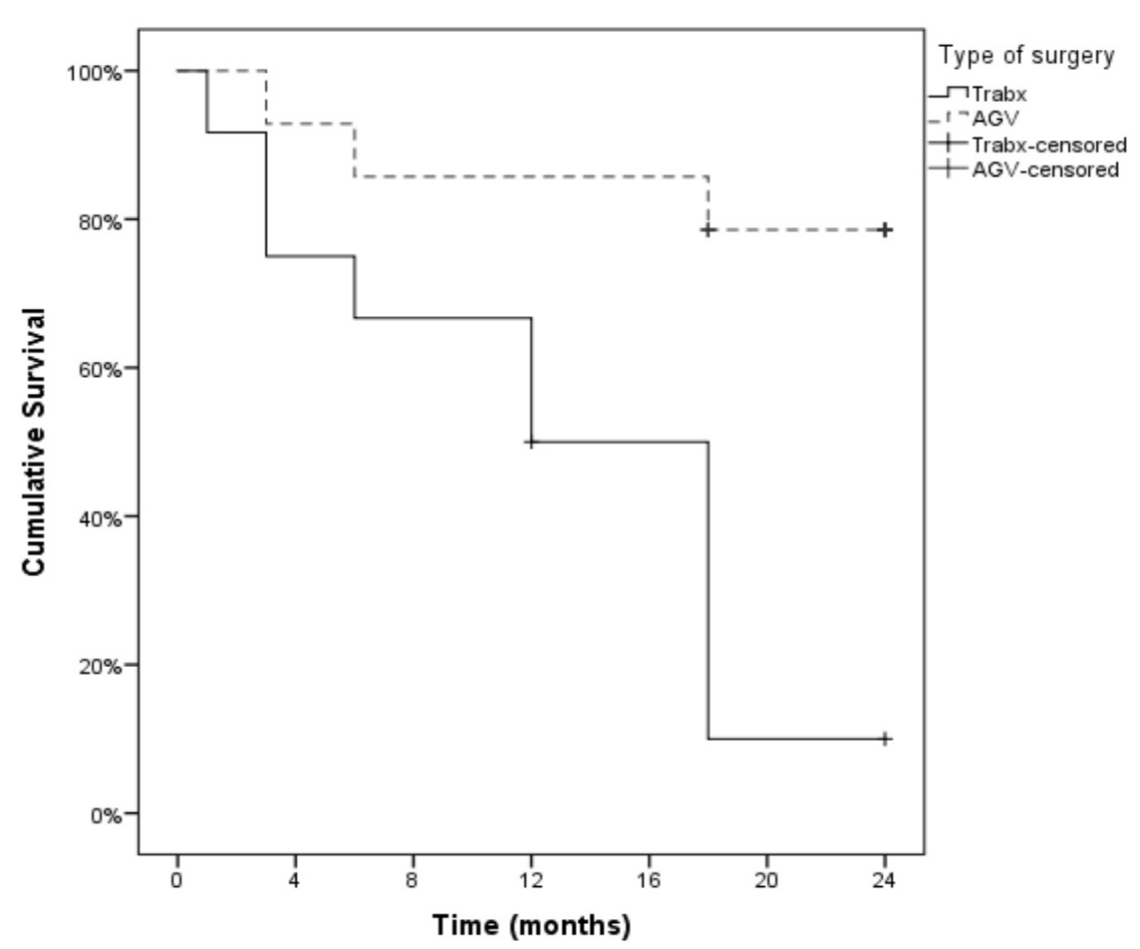
Kaplan–Meier survival curve for trabeculectomy (Trab) (solid line) and Ahmed glaucoma valve (AGV) (dotted line) surgeries for Fuchs heterochromic iridocyclitis-associated glaucoma in this study.

Patients in the AGV group needed fewer glaucoma medications at the final follow-up (P= 0.041). Kaplan–Meier survival curves for the two groups are shown in Figure 1. The estimated mean survival time of the surgery was 20.8 months for those in the AGV group and only 12.7 months for those in the trabeculectomy group (P= 0.002). The reason for failure of trabeculectomy was bleb fibrosis. Five patients (37.5%) in the AGV group experienced an early hypertensive phase.

Log-rank P = 0.002. Estimated mean survival time of trabeculectomy was 12.7 months (95% confidence interval, 8.5–16.9). Estimated mean survival time for glaucoma drainage device implant surgery is 20.8 months (95% confidence interval, 17.2–24.4).

Triamcinolone had no impact on IOP (P= 0.320). The most frequent complication in both groups was hyphema ( Table 3). In total, five patients in the trabeculectomy group (41.6%) and three patients in the AGV group (21.4%) developed hyphema (P= 0.292) which could be managed conservatively. There was one patient in the AGV group and three in the trabeculectomy group that exhibited established choroidal effusions that had to be drained. The anterior cell reaction did not exceed 0.5 during the preoperative or postoperative exam and there were no significant difference between the AGV and trabeculectomy groups (p=0.871 and 0.9, respectively). One patient in each group developed endophthalmitis. The endophthalmitis in the patient that underwent AGV was preceded by tube exposure. The patient underwent vitrectomy and the device was removed. A new AGV was implanted in the infranasal location in the same session. Although the endophthalmitis in trabeculectomy could be controlled by an injection of intravitreal antibiotics and a corticosteroid (vancomycin (25 mg in 0.5 ml), ceftazidime (100 mg in 0.5 ml) and dexamethasone (6 mg in 0.25 ml) injected as a bolus), a glaucoma drainage device was needed. In the AGV group, two patients experienced endothelial touch, and one of them underwent tube shortening due to early corneal decompensation. Hypotony was observed in two cases in the trabeculectomy group in the early postoperative period, which resolved without a surgical intervention within 1 month. There was no significant difference between the rate of complications between the two groups ( Table 3). None of the listed complications were significant factors for surgical failure in AGV or trabeculectomy. A cataract extraction was indicated in five patients in the trabeculectomy group and in only one patient in the AGV group. The mean time between trabeculectomy and cataract surgery was 9.1±4.3 months.

**Table 3.** Postoperative complications in trabeculectomy and Ahmed glaucoma valve (AGV) groups.

Variable	Trabeculectomy	AGV	P-value
Early hypotony (within 3 months of surgery)	2	none	0.31
Late hypotony (over 3 months after surgery)	none	none	
Hyphema	5	3	0.292
Choroidal effusion	3	1	0.246
Corneal decompensation	0	1	0.213
Endophthalmitis	1	1	
Tube exposure (AGV only)	N/A	2	N/A
Tube-cornea touch (AGV only)	N/A	2	N/A
Bleb leakage (trabeculectomy only)	3	N/A	N/A
Diplopia	0	0	

N/A, not applicable.

Raw data collected from all study participants Click here for additional data file. Copyright: © 2018 Esfandiari H et al. 2018 Data associated with the article are available under the terms of the Creative Commons Zero "No rights reserved" data waiver (CC0 1.0 Public domain dedication).

## Discussion

In this retrospective study, we evaluated the outcome of two common surgeries for FHIC-associated glaucoma, a valved tube shunt (AGV) and trabeculectomy with MMC. Although FHIC is rare, occurring in about 0.00105% of the population ^2, 3^, and the course is typically mild, almost 50% of patients with FHIC develop glaucoma ^12– 14, 20^ and require aggressive management. We found that AGV had a significantly higher success rate than trabeculectomy, confirming our hypothesis. Unexpectedly, patients also needed fewer glaucoma medications in AGV, while the complication rate was similar.

Most glaucoma patients exhibit open-angle configuration on gonioscopy. Decreased outflow is instead caused by inflammatory cells, fibrotic changes of the trabecular meshwork, and long-term steroid use ^13– 15^. The management of FHIC-associated glaucoma is challenging ^21^. In a study by Liesegang, 66% of patients with FHIC-associated glaucoma needed surgical intervention and did so earlier in life than individuals with primary open-angle glaucoma ^16^. Laser trabeculoplasty is contraindicated because it can exacerbate the inflammation, cause bleeding from neovascularization of the angle and induce peripheral anterior synechiae ^22^.

When the uveitis is only mildly active, a trabeculectomy can be performed to quickly lower IOP, including in FHIC ^23^ even though the risk of bleb failure is relatively high in uveitic glaucoma ^24^. Although the trabeculectomy success rate in uveitis is above 50% at 5 years ^25, 26^, it is lower the one reported for epibulbar glaucoma drainage implants even in primary open-angle glaucoma ^27^

Although FHIC is not typically characterized by severe inflammation, trabeculectomy outcomes have been reported to be worse ^13, 14^. The high rate of hyphema in our series likely contributed to this because blood can reduce the bleb size in trabeculectomy ^28, 29^ but not in tube shunts. Hyphema commonly occurs in FHIC because of the angle neovascularization in FHIC ^30^ and rupture of these fragile vessels following IOP reduction ^5^. Another risk factor for bleb failure is the higher rates of cataract extraction in T. Cataract formation or progression is common even after uneventful trabeculectomy with a range of 6 to 58 % ^30^. The fact that trabeculectomy tends to enhance cataract progression while cataract extraction can reduce the success rate of trabeculectomy limits its use in the management of glaucoma in FHIC. In our study, five out of ten patients required cataract surgery. The high rate of cataract formation after trabeculectomy appears to be an under-reported risk of failure of trabeculectomy in FHIC-associated glaucoma. For this reason, same-session cataract removal should be considered because modern phacoemulsification at the time of glaucoma surgery may have only a negligible impact on IOP outcomes ^31^

The reported intermediate success rate for glaucoma drainage devices in uveitic glaucoma is between 66% and 85% ^32– 34^. In a study by Tan *et al*., the short- and long-term success rate of non-valved Baerveldt implants in uveitic glaucoma was 89% and 75%, respectively ^35^. In another study, Satana *et al*. used valved Ahmed implants in 14 patients with uveitic glaucoma secondary to Behcet disease and reported the cumulative probability of surgical success rate of 90.9% at 18 months follow-up ^33^. Kwon *et al.* examined the outcome of AGV implantation in 28 patients with uveitic glaucoma including FHIC and reported a success rate of 75% during the 2-year follow-up ^36^. Voykov and colleagues assessed the short and intermediate-term success rate of AGV implantation in 17 patients with FHIC- associated glaucoma ^37^. Qualified success defined as 6 mmHg ≤ IOP ≤21 mmHg was achieved in 58.3% of patients after 1 year and only 38.4% after 3 years, although 88% of patients had conjunctival scarring from prior procedures, a known risk factor ^38^. This may also explain the rate of complications (23% tube exposure, 23% device exposure, 6% endophthalmitis, 6% diplopia, 11.7% hypotony) in the mentioned study.

Our results indicate that primary AGV has a higher cumulative probability of success in FHIC. Regardless, consistent with prior studies ^34, 39^, our complication rate was high. This highlights how challenging and unpredictable uveitis is, even though FHIC is a relatively mild form of uveitis. Given the high rate of complications and the patients’ age, microincisional procedures should be considered for FHIC that have been proven to be safe and effective in other mild-to-moderate forms of uveitic glaucoma ^40, 41^, and appropriate for a range of glaucoma severity ^42^. The occurrence of a hypertensive phase in the current study is lower than the 47% incidence rate reported by Voykov *et al.* ^37^. The lower rate could partially be explained by the modulation of encapsidation and inflammation by triamcinolone used in several patients here, although contradictory results have been reported ^43, 44^. Bleb vascularity is a recently identified risk factor for bleb failure ^27^ that we did not examine here. An intensified treatment for this problem can include bevacizumab, which can be used subconjunctivally ^45^ instead of intravitreally ^46^. Another explanation for the lower incidence of early hypertension may be the start of aqueous suppressant to reduce fibroblast stimulation from stretch and cytokines ^47^.

Limitations of this study are the relatively small patient number dictated by the overall rareness of FHIC, a retrospective design and the use of triamcinolone in some patients. Additionally, in this retrospective study the anterior cell reaction was not assessed systematically and objectively enough to test for a formal correlation to the outcome of surgeries. While the anterior chamber cell reaction in FHIC is not as prominent as in other uveitic glaucomas and remained at or below a grading of 0.5, it would be interesting to examine this aspect in more detail to try to understand why AGV patients did better

In conclusion, this study shows that Ahmed glaucoma drainage devices are superior to trabeculectomy in FHIC-associated glaucoma. The relatively high complication rate is consistent with prior reports and highlights the considerable risk associated with this relatively mild form of uveitis.

## Data availability

The data referenced by this article are under copyright with the following copyright statement: Copyright: © 2018 Esfandiari H et al.

Data associated with the article are available under the terms of the Creative Commons Zero "No rights reserved" data waiver (CC0 1.0 Public domain dedication). 

**Dataset 1. Raw data collected from all study participants.** DOI: http://doi.org/10.5256/f1000research.15244.d207417 ^48^.
