# Supplementary material for: Fuchs heterochromic iridocyclitis-associated glaucoma: a retrospective comparison of primary Ahmed glaucoma valve implantation and trabeculectomy with mitomycin C
Source: F1000Res. 2018 Oct 15;7:876. Originally published 2018 Jun 22. [Version 2] doi: 10.12688/f1000research.15244.2 (PMC6198260; doi:10.12688/f1000research.15244.2)
Supplement: Peer review history

# Peer review: Referee response for version 2

**DOI:** 10.5256/f1000research.18137.r39467

**Published:** 2018-10-22

**Authors:** Naveed Nilforushan

**Affiliations:** 1 Eye Research Center, Rassoul Akram Hospital, Iran University of Medical Sciences, Tehran, Iran

The authors have properly addressed all comments, so it is now acceptable.

I have read this submission. I believe that I have an appropriate level of expertise to confirm that it is of an acceptable scientific standard.

# Peer review: Referee response for version 1

**DOI:** 10.5256/f1000research.16605.r37324

**Published:** 2018-08-29

**Authors:** Naveed Nilforushan

**Affiliations:** 1 Eye Research Center, Rassoul Akram Hospital, Iran University of Medical Sciences, Tehran, Iran

General comment:

 The authors are trying to compare the results of Trabx and AGV in FHI patients, but the main concern and cofounding factor are the significant difference between the rates of phaco surgery during the follow up which is known as a risk factor for the failure of Trabx. This effect could be more prominent when another risk factor such as chronic uveitis is present. The main cause of such difference between 2 groups, in terms of success, with sharp downward slope after month 6 is most probably related to cataract surgery. This needs to be discussed more.

 Specific comments:

1. The hypothesis mentioned in the last paragraph of introduction which has been stressed again in the first paragraph of the discussion needs more justification and explanation. What does it mean: “having lower failure rate but at the expense of higher average pressure in AGV” According to the cited article “ *Lower IOPs were noted for the trabeculectomy group during the first year. With longer follow-up, the IOPs and the cumulative probabilities of success were comparable between the two groups*”. Higher success rate for AGV in other studies such as TVT was due to higher rates of hypotonia in Trabx which was not the cause of failure in the present study.
2. Combined glaucoma surgery and phaco was among the exclusion criteria, but 2 cases in Trabx have had a combined surgery. These cases should have been excluded.
3. By considering which criteria, in some cases Trabx and in others AGV were performed?
4. The definition for hypertensive phase is not precise and cannot differentiate those cases with non functioning tube from hypertensive phase. Please give some explanations and give the rate of hypertensive phase.
5. How many surgeons did perform those surgeries? 
6. Were there cases that needed postop needling or subconjunctival antifibrotic injections?
7. Was the grading of inflammation on those with failed surgery during follow up different from the others?
8. Although the following 2 references have used old fashion type of surgery (No antifibrotic or 5FU) but still according to reference 26 and 27 the 5 year success rate of Trabx was 78% and 67% respectively. This has been mentioned less than 30% in the article. Please correct or give explanation.

I have read this submission. I believe that I have an appropriate level of expertise to confirm that it is of an acceptable scientific standard, however I have significant reservations, as outlined above.

# Response

**Published:** 2018-10-08

**Authors:** Nils Loewen

**Affiliations:** University of Pittsburgh Medical Center, USA

**General comment:**

 The authors are trying to compare the results of Trabx and AGV in FHI patients, but the main concern and confounding factor are the significant difference between the rates of phaco surgery during the follow up which is known as a risk factor for the failure of Trabx. This effect could be more prominent when another risk factor such as chronic uveitis is present. The main cause of such difference between 2 groups, in terms of success, with a sharp downward slope after month 6 is most probably related to cataract surgery. This needs to be discussed more.

**Authors: ** Thank you for this suggestion. We have added the following: “Another risk factor for bleb failure is the higher rates of cataract extraction in T. Cataract formation or progression is common even after uneventful trabeculectomy with a range of 6 to 58 %. The fact that trabeculectomy tends to enhance cataract progression while cataract extraction can reduce the success rate of trabeculectomy limits its use in the management of glaucoma in FHIC.”

**Specific comments:**

**1.  **  The hypothesis mentioned in the last paragraph of introduction which has been stressed again in the first paragraph of the discussion needs more justification and explanation. What does it mean: “having lower failure rate but at the expense of higher average pressure in AGV” According to the cited article “Lower IOPs were noted for the trabeculectomy group during the first year. With longer follow-up, the IOPs and the cumulative probabilities of success were comparable between the two groups”. The higher success rate for AGV in other studies such as TVT was due to higher rates of hypotonia in Trabx which was not the cause of failure in the present study.

**Authors:** We appreciate and agree and have deleted this statement. We now state: “There is a paucity of literature regarding the best initial surgical approach in the management of FHIC-associated glaucoma. The purpose of this study was to compare the outcomes of the two most common surgical interventions, glaucoma drainage device implantation, and trabeculectomy, for glaucoma caused by FHIC.”

**2. **      Combined glaucoma surgery and phaco were among the exclusion criteria, but 2 cases in Trabx have had a combined surgery. These cases should have been excluded.

**Authors:** Thank you for pointing this out. We have corrected the description of exclusion criteria. They consisted of prior glaucoma surgery, ocular or systemic comorbidities that could affect the procedure and study outcomes including immunodeficiency, connective tissue disease and uncontrolled diabetes. Patients were not formally matched across demographics.

**3.  **     By considering which criteria, in some cases, Trabx and in others AGV were performed?

**Authors:** There were no strict criteria. Based on the surgeon’s preference and comfort as well as patients’ decision, one of these procedures was performed.

**4.**       The definition for the hypertensive phase is not precise and cannot differentiate those cases with a non-functioning tube from the hypertensive phase. Please give some explanations and give the rate of hypertensive phase.

**Authors:** We added the following in the Methods and Results section, respectively, to be more clear about the hypertensive phase: “A hypertensive phase following AGV implantation was defined as an IOP rising above 21 mmHg during the first 3 months with or without medications after an initial IOP that was lower than 21 mmHg.”  

 “The reason for trabeculectomy failure was bleb fibrosis. Five patients (37.5%) in the AGV group experienced an early hypertensive phase. All patients in the AGV group had early postoperative IOPs of less than 21 mmHg indicating that all valved implants were functioning properly.”

**5. **      How many surgeons did perform those surgeries?

**Authors:** We added to Methods: “Three surgeons performed the procedures.”

**6.    **   Were there cases that needed postop needling or subconjunctival antifibrotic injections?

**Authors:** We added to Results: “Eight trabeculectomy patients needed bleb needling with MMC.  Needling was done during the postoperative period for impending failure from a contracting bleb. Thirty minutes after injecting 0.1 ml of 0.02% Mitomycin-C into the bleb-adjacent subtenon space a 27 gauge needle was used to reform the bleb and dissect adhesions at the slit lamp.”

**7.**       Was the grading of inflammation on those with failed surgery during follow up different from the others?

**Authors:** We now mention in the Discussion: “Additionally, in this retrospective study the anterior cell reaction was not assessed systematically and objectively enough to test for a formal correlation to the outcome of surgeries. While the anterior chamber cell reaction in FHIC is not as prominent as in other uveitic glaucomas and remained at or below a grading of 0.5, it would be interesting to examine this aspect in more detail to try to understand why AGV patients did better.”  

**8.     **  Although the following 2 references have used old fashion type of surgery (No antifibrotic or 5FU) but still according to reference 26 and 27 the 5 years success rate of Trabx was 78% and 67% respectively. This has been mentioned less than 30% in the article. Please correct or give an explanation.

**Authors: **Thank you. We changed that paragraph to now read: “When the uveitis is only mildly active, a trabeculectomy can be performed to quickly lower IOP, including in FHIC 24 even though the risk of bleb failure is relatively high in uveitic glaucoma 25. Although the trabeculectomy success rate in uveitis is above 50% at 5 years 26, 27, it is lower the one reported for epibulbar glaucoma drainage implants even in primary open-angle glaucoma 28.

# Peer review: Referee response for version 1

**DOI:** 10.5256/f1000research.16605.r35657

**Published:** 2018-08-13

**Authors:** Syril K. Dorairaj, Marcos Pereira Vianello

**Affiliations:** 1 Department of Ophthalmology, Mayo Clinic, Jacksonville, FL, USA; 2 the Glaucoma and Cataract Department, Medical Sciences Eye Institute, Belo Horizonte, Brazil

This is an interesting study where authors retrospectively compared the safety and efficacy of primary trabeculectomy with Mitomycin C and Ahmed glaucoma valve (AGV) implantation in patients with Fuchs heterochromic iridocyclitis (FHIC)-related glaucoma. Esfandiari et al hypothesized that Ahmed glaucoma drainage devices, would have a lower failure rate compared to trabeculectomy and based on their results, authors successfully conclude that Primary AGV implantation had a higher success rate than trabeculectomy, with patients also needing fewer medications for the management of FHIC-associated glaucoma. 

 Though there is a general trend towards utilizing minimally invasive angle based surgeries, authors have done a timely job in evaluating trabeculectomy and AGV since there is greater risk of failure of glaucoma surgery consequent of trabecular meshwork fibrosis and steroid response in FHIC associated glaucoma. This study gives insight into designing a randomized control trials comparing device-assisted trabeculectomy with conventional trabeculectomy and glaucoma drainage device in the future.

We have read this submission. We believe that we have an appropriate level of expertise to confirm that it is of an acceptable scientific standard.
